# A Low-Cost Flexible Perforated Respiratory Sensor Based on Platinum for Continuous Respiratory Monitoring

**DOI:** 10.3390/mi13101743

**Published:** 2022-10-14

**Authors:** Lu Cao, Zhitong Zhang, Junshi Li, Zhongyan Wang, Yingjie Ren, Qining Wang, Dong Huang, Zhihong Li

**Affiliations:** 1 National Key Laboratory of Science and Technology on Micro/Nano Fabrication, Beijing 100871, China; 2 College of Engineering, Peking University, Beijing 100871, China; 3 School of Integrated Circuits, Peking University, Beijing 100871, China

**Keywords:** wearable device, respiratory sensor, temperature, sleep monitoring

## Abstract

Monitoring sleep conditions is of importance for sleep quality evaluation and sleep disease diagnosis. Accurate respiration detection provides key information about sleep conditions. Here, we propose a perforated temperature sensor that can be worn below the nasal cavity to monitor breath. The sensing system consists of two perforated temperature sensors, signal conditioning circuits, a transmission module, and a supporting analysis algorithm. The perforated structure effectively enhances the sensitivity of the system and shortens the response time. The sensor’s response time is 0.07 s in air and sensitivity is 1.4‰°C^−1^. The device can achieve a monitoring respiratory temperature range between normal room temperature and 40 °C. The simple and standard micromachining process ensures low cost and high reproducibility. We achieved the monitoring of different breathing patterns, such as normal breathing, panting, and apnea, which can be applied to sleep breath monitoring and exercise information recording.

## 1. Introduction

Breath is a basic physiological activity reflecting physical and mental states of the human body [1]. For example, rapid breath often indicates stress, panic, or fear, and high exhaled gas temperatures may indicate respiratory inflammation [2,3]. Moreover, many physiological and psychological problems, such as stress, anxiety, chronic obstructive pulmonary disease, and post-traumatic stress disorder, can be regulated or alleviated by respiratory treatment [4,5]. 

With increasing social pressures, the incidence of sleep disorders is getting higher and higher, often accompanied by abnormal respiratory system symptoms. Obstructive Sleep Apnea-Hypopnea Syndrome (OSAHS) is the most common sleep respiratory disorder [6,7,8]. It damages the cardiovascular system and may cause hypertension, aggravation of respiratory failure, arrhythmia, and even sudden death [9]. Continuous hypoxia caused by apnea or hypopnea may lead to myocardial infarction or cerebral infarction. In addition, according to relevant investigations and studies, the incidence increases with age [10,11]. Therefore, continuous and accurate respiratory monitoring is important in both daily and clinical applications [12].

At present, the sensing mechanisms of respiratory sensors mainly include temperature, humidity, airflow, stress and strain [13,14,15]. Polysomnography (PSG) is commonly used in the sleep examination department of hospitals to manifest sleep respiratory signals and other electrophysiological signals [16]. These devices use intubation-type nasal airflow pressure and temperature monitoring modules that invade the nasal cavity and cause strong discomfort. Additionally, thorax and abdomen breath monitoring using a piezoelectric belt is susceptible to noise caused by limb movements and affects normal breath. There are also some indirect measurement methods, such as 3D image sensing, ultrasonic sensing, and fiber-optic sensing, which can be used to monitor respiration by detecting chest movement. However, these measurement schemes are susceptible to body movements [17,18,19,20,21]. Presently, some respiratory monitoring devices use face-mask nasal airflow detection devices, which are too large and cause discomfort [22]. Jin et al. used a piezoresistive MEMS sensor (connected through a medical nasal cannula) to obtain breathing signals for detecting apnea [23]. Shinya Kano et al. proposed an all-painting process to produce a respiration sensor made from a humidity-sensitive nanoparticle (NP) film and a graphite trace [24]. Tong Zhang et al. describe how polymer humidity sensors with humidity-sensitive polyelectrolytes perform ultrafast responses for respiration monitoring. Asadnia et al. have developed a polymeric airflow sensor based on nanocomposites of vertically grown graphene nanosheets (VGNs) with polydimethylsiloxane (PDMS) and have explored their applications in monitoring human respiration. Xue Feng et al. built skin-like hybrid integrated circuits (SHICs) with stretchable temperature sensors and commercial chips for long-term respiration monitoring [25]. However, the response and recovery times of the device are relatively long. Fiber Bragg grating (FBG) is also utilized to monitor respiration, which has the advantages of small size and anti-electromagnetic interference. However, a hygroscopic coating and a special optical demodulator are additionally required to read the information [26,27]. Y. Liu et al. introduced an ultrathin respiration sensor based on the thermal convection effect. The device features a filamentary fractal design with a gold heating electrode and a mini sensor with high thermal sensitivity [28]. Tao Jiang et al. introduced a wearable hot-film/calorimetric breath sensing system composed of a hot-film senor in the center and two calorimetric sensors on two sides [29]. However, the power consumption of this kind of sensor is large. Low thermal conductivity for the substrate is significant for maintaining unnecessary heat loss. The thermal conductivity of the silicon substrate is high; therefore, it needs to be suspended. The photolithography process for perforated or suspended structures is more complicated, and the cost is higher. Additionally, the suspended metal wires are easily destroyed. The perforated PI structure has sufficient mechanical strength and low thermal conductivity. 

**Figure 1:**
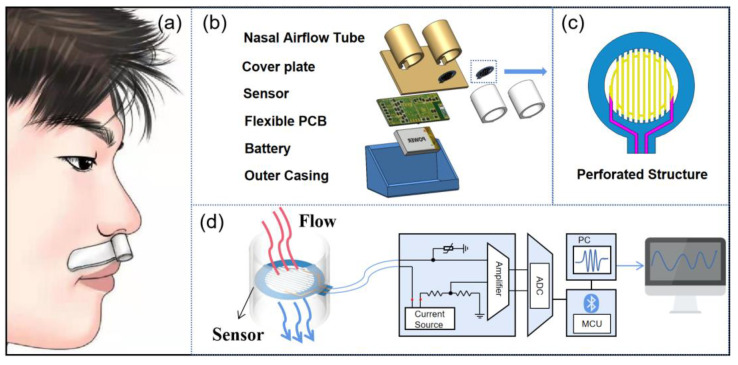
Schematic view of the proposed device. (**a**) Schematic diagram of wearing method. (**b**) In depth schematic illustration of the wireless sensing platform, enclosure architectures and battery. (**c**) Geometry of the perforated respiratory sensor. (**d**) Schematic representation of the sensor connected with measuring circuit and PC. sensor.

This paper reports a perforated respiratory sensing system, which is composed of two flexible perforated respiratory sensors (fPRS), a signal processing circuit, a lithium battery, and enclosure architectures. The perforated respiratory sensor effectively improves the gas convection efficiency and the thermal sensitivity. As shown in Figure 1a, the device is placed below the nose, where it is able to accurately sense the human breath status. The signal processing circuit is used for signal acquisition, conditioning and transmission, as shown in Figure 1d. At the same time, it can also transmit the breathing signal to the external device for real-time display. With the algorithm design, it can realize the diagnosis of obstructive apnea, hypopnea, or other respiratory diseases.

## 2. Experimental Section

### 2.1. Design of the Sensor

Platinum (Pt) is a material with stable physical and chemical properties, and is compatible with various fabrication processes [30,31]. The resistance-temperature characteristic of Pt shows good linearity and a fast thermal response. Moreover, its good electrical properties and thermal conductivity make it sensitive to temperature changes, thereby helping to reduce the drive current. The above advantages prove that Pt is a suitable material for respiratory temperature sensors with high-precision measurement. The choice of low thermal conductivity for the substrate is significant to maintaining unnecessary heat loss around the substrate and the sensor. Therefore, Polyimide (PI) is selected as the substrate material.

Figure 1c provides an image of the perforated respiratory sensor. The perforated structure of the substrate can simplify the design of the mask and control the sputtering graphics, which can be directly processed. It is of great significance to improve the alignment accuracy of the mask. As shown in Figure 1b, the small rounded fPRS can be installed in the short nasal breathing tube, which is very light to wear and does not affect normal physical activities. The warm air exhaled from the nostril can pass through the perforated structure and fully contact the platinum temperature-sensitive layer, which effectively improves the sensitivity of the sensor. 

In general, the calculation formula of metal resistance is as follows:

(1) $$R=\rho \frac{L}{S}$$

where *ρ* is the metal resistance, *L* is length of resistance, and *S* is the cross-sectional area of resistance. 

According to the Fuchs–Sondheimer (FS) and Mayadas–Shatzkes (MS) models, metals with a smaller mean electron free path (EMFP) exhibit a size effect for smaller dimensions [32]. An inverse relationship between film thickness and electrical resistivity has been commonly demonstrated with the FS surface scattering and MS grain boundary scattering models [33,34]. According to the FS model, the resistivity of thin film is given by:

(2) $$\rho =\rho_{bulk}\left[1-\frac{3}{2k}\left(1-p\right)\int_{1}^{\infty }(\frac{1}{t^{3}}-\frac{1}{t^{5}}\right)\frac{1-e^{-kt}}{1-pe^{-kt}}dt{]}^{-1},\;k=\frac{d}{\lambda }$$

where *d* is the film thickness, *k* is the EMFP, *λ* is the intrinsic electron mean free path and *p* is the surface scattering factor. As the platinum film thickness approaches the EMFP (about 30 nm), a strong increase in resistivity is observed [35].

Considering that the inner diameter of the nose of an adult is generally less than 1 cm, the design sensor diameter is 8 mm. The geometry is designed with a line width of 200 μm and spacing of 200 μm as the sensing area. The metal platinum film thickness of the sensor is 50 nm.

### 2.2. Fabrication

**Figure 2:**
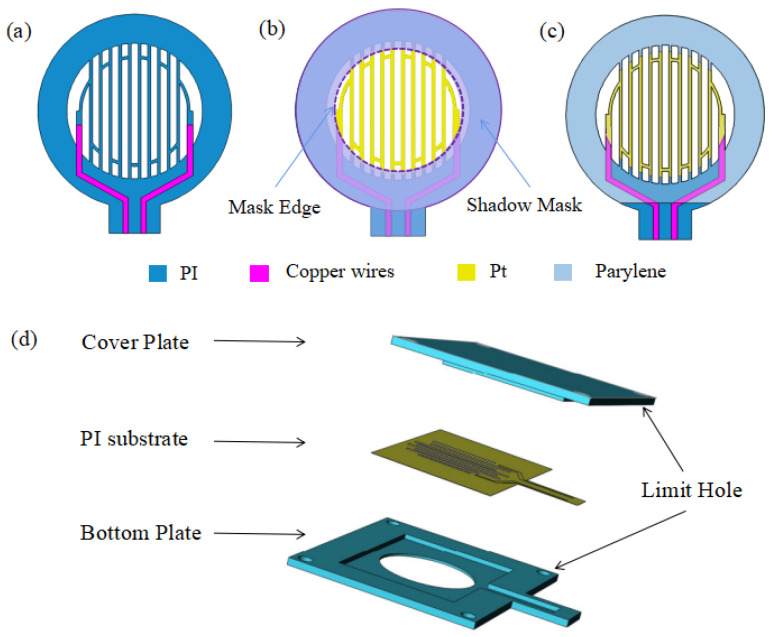
Fabrication processes of the perforated respiratory sensor. (**a**) Print two copper leads on PI substrate and laser cut the target pattern. (**b**) Pt deposition on PI substrate with shadow mask. (**c**) Parylene-C deposition on the surface. (**d**) The shadow mask is designed as a bottom plate with a graphic groove and a cover plate with limit holes.

Figure 2 shows the manufacturing process of the perforated respiratory sensor. Firstly, two copper leads are patterned on the PI substrate via the standard flexible printed circuit board (fPCB) process. The mesh-hollowed shape is made by performing laser-cutting, a standard process for fPCB, forming a perforated structure as shown in Figure 2a. Then, it is soaked in ethanol and cleaned ultrasonically for 10 min to remove organic impurities on the substrate. Afterwards, it is treated with oxygen plasma cleaning to remove the insoluble organic impurities on the surface and strengthen the adhesion of Pt. Finally, the Pt layer is sputtered with a shadow mask for pattering of the thermo-sensitive layer. The dashed line is the edge of the shadow mask, as shown in Figure 2b.

It is worth noting that the perforated pattern on the substrate is specially designed to tolerate the edge diffusion and misalignment during the sputtering process, ensuring good sensor consistency. With this design, the path of the series resistor will always remain unchanged when process deviation occurs. Moreover, a self-alignment structure is designed to further increase the alignment accuracy. As shown in Figure 2d, the mask is designed as a bottom plate with a graphic groove and a cover plate, which can be combined together and fixed by the limit holes at the four corners to ensure the stability of the mask during the sputtering process.

In order to avoid the influence of air humidity and mechanical damage on the sensor in use, Parylene-C is encapsulated by Chemical Vapor Deposition (CVD). By configuring the deposition rate, the 0.3 μm-thickness Parylene-C can be controlled to achieve a good packaging effect as shown in Figure 2c.

### 2.3. Simulation

**Figure 3:**
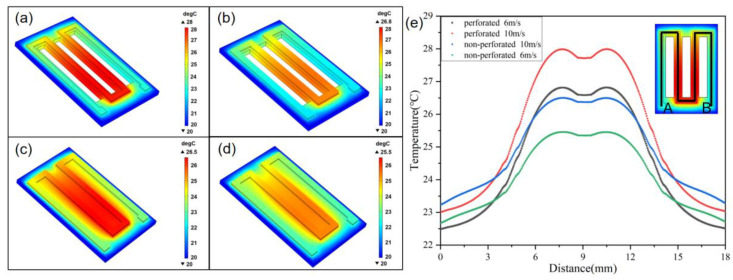
Comparison of the heat transfer properties between the non-perforated sensor and perforated sensor. (**a**) The temperature distribution of the perforated sensor at a respiratory airflow speed of 10 m/s and (**b**) at a speed of 6 m/s. (**c**) The temperature distribution of the non-perforated sensor at a speed of 10 m/s and (**d**) at a speed of 6 m/s. (**e**) The temperature distributions from point A to B for non-perforated and perforated sensors at different respiratory airflow rates.

The thermodynamic simulation of the device is carried out using finite element analysis software (COMSOL 5.4) to prove the excellent thermal conductivity of the fPRS. The environment of the model is set as air; the substrate and heat-sensitive materials are PI and Pt, respectively. The relevant parameters are set to simulate the temperature change caused by breathing. It is assumed that the ambient air temperature far away from the sensor is 20 °C and the respiratory air temperature is 30 °C. Set a voltage of 0.01 V on one end of the model and ground on the other end. Set the heat transfer module to an external forced convection, and set 6 m/s and 10 m/s, respectively, to simulate slow breathing and fast breathing. The same conditions are used to simulate a non-perforated structure and perforated structure, respectively. The temperature range of the perforated sensor is 20 °C to 26.8 °C at a respiratory airflow speed of 6 m/s, with a maximum temperature of 28.0 °C at a speed of 10 m/s, as shown in Figure 3a,b. The temperature range of the non-perforated sensor is 20 °C to 25.5 °C at a speed of 6 m/s, and the maximum temperature is 26.5 °C at a 10 m/s speed, as shown in Figure 3c,d. Figure 3e shows the temperature distributions from point A to B for non-perforated and perforated sensors at different respiratory airflow rates, proving that the perforated sensor has a larger temperature dynamic range. By comparison, it can be found that the perforated sensor has a higher temperature response, indicating that it is more sensitive to the temperature change of breathing.

## 3. Results and Discussion

### 3.1. Metrological Characterization

**Figure 4:**
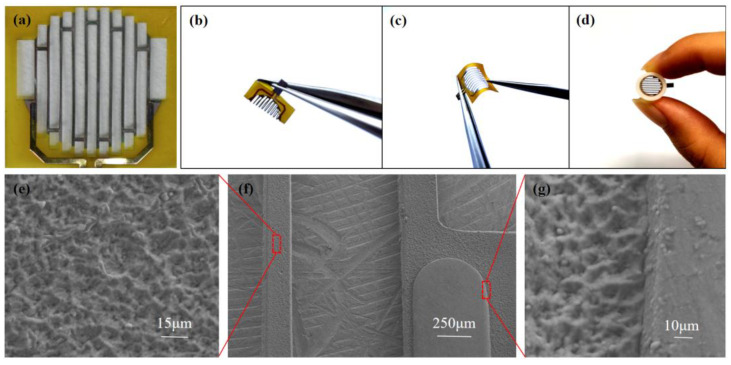
(**a**) Microscopic image of the perforated respiratory sensor. (**b**) 180-degree bend and (**c**) 90-degree bend. (**d**) Integrated picture. (**e**,**f**) SEM image of the perforated respiratory sensor. (**g**) SEM image of the connection between copper wire and platinum.

The original impedance of the fPRS is about 14 kΩ, and there is good consistency between different sensors. Through observation under a microscope, as shown in Figure 4a, the appearance is complete and uniform, and the edges are straight and neat. The sputtered Pt film has no cracks, and the width and thickness are uniform, as shown in Figure 4e–f. The connection between the copper wire and platinum is firm, as shown in Figure 4g.

We attached the sensor to the nasal air duct, as shown in Figure 4d. The material of the tube is Acrylonitrile Butadiene Styrene (ABS) which is non-toxic and harmless. These nasal air ducts can prevent the influence of the external airflow. Simple multiple mechanical tests are performed on the fPRS including 180-degree and 90-degree bending, as shown in Figure 4b,c, showing good flexibility. The flexibility property of the sensor prevents damage during insertion into the nasal air duct.

**Figure 5:**
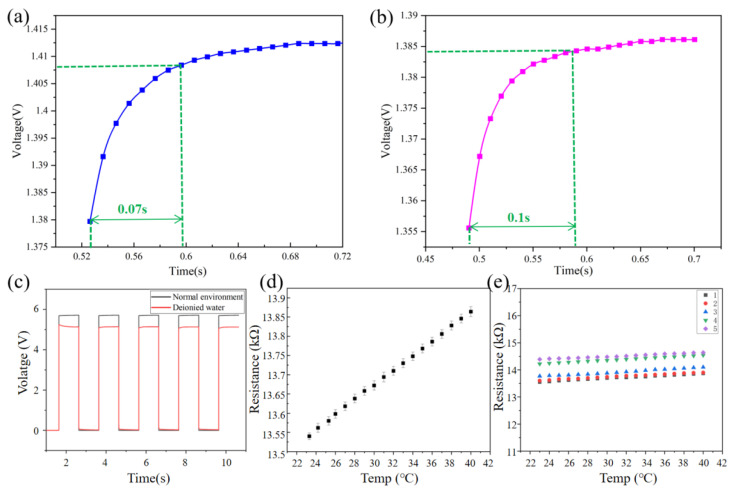
(**a**) Test the response time of the sensor in air. Test of constant current in Electrochemical Workstation. (**b**) Test the response time of the sensor in deionized water. (**c**) Pulse test in air and deionized water. (**d**) Repeatability of temperature test of the sensor. (**e**) Repeatability of temperature test of five sensors.

We tested the response time of the sensor under different conditions in the electrochemical workstation (PGSTAT302N). As the actual operating condition is 100 μA, the test condition is to implement a 100 μA direct current at one end and ground the other end. The test result is shown in Figure 5a. According to the calculation of the amplitude, rising to 90% of the maximum, the response time of the sensor is 0.07 s. We know the respiratory rate of humans is 0.2–0.5 Hz. The response time is much smaller than the human breathing period, indicating that the perforated respiratory sensor is sufficiently fast when monitoring breathing signals. In order to verify the performance of the sensor to isolate the influence of humidity, the sensor was placed in deionized water; the response time is 0.1 s, as shown in Figure 5b. The impulse test is performed by applying a pulse current at intervals of 1 s. As shown in Figure 5c, the response time is small and there is almost no delay. The performance of the sensor fully meets the response time requirements of respiratory monitoring. A respiratory temperature sensor and standard high-precision temperature module are placed close to one another in a heating box. The temperature of the heating box was set from 23 °C to 40 °C with a step of 1 °C, and the resistance of the sensor was measured after the temperature stabilized. Sensors were tested five times and the mean and standard error of the mean were calculated. Figure 5d shows that the repeatability is good. The same method was used to test five sensors and more consistent results were obtained, as shown in Figure 5e.

### 3.2. System Set-Up and Calibration

**Figure 6:**
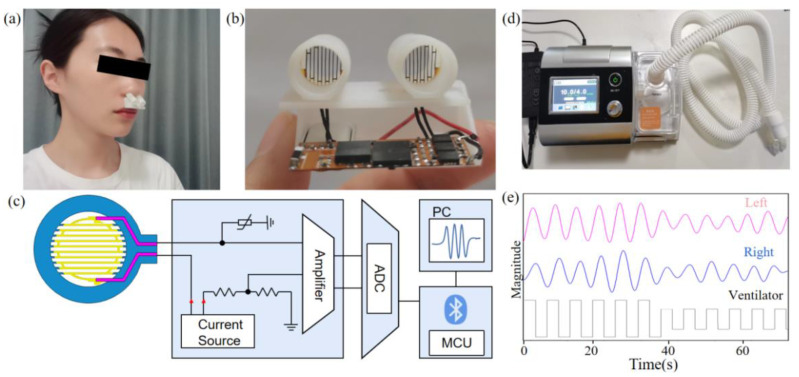
(**a**) Photograph of the device worn below the nose. Subject: 23 years of age, female, nonprofessional healthy volunteer. (**b**) Device with wires is connected to external circuit modules. (**c**) Architecture of signal acquisition circuit. (**d**) Experimental setup for respiration simulation. (**e**) Monitoring results of simulated respiratory signals.

We designed a circuit acquisition module with a Bluetooth function, thereby improving the comfort of breathing monitoring. The way of wearing the respiratory sensor is shown in Figure 6a, greatly reducing the discomfort of the subjects. The sensors can be connected to external circuit modules by flexible wires, as shown in Figure 6b. A lithium battery is also encapsulated by the case. The geometric dimensions of the shell are 38 cm in length, 10 cm in width, and 15 cm in height, respectively, and the inclination angle is 40 degrees. The device does not penetrate the nostrils, and it can be reused after alcohol sterilization.

The architecture of the circuit is shown in Figure 6c, including the fPRS, current source, amplifier, analog-to-digital converter (ADC), and micro control unit (MCU) board with Bluetooth. The power module includes a 100 μA constant current source chip. The constant current is added to the respiratory sensor and a thermistor, and the voltage of the sensor and thermistor is differentially amplified using an amplifier. The resistance of the sensor can be obtained by measuring the output voltage. The thermistor can monitor the external ambient temperature, and after differential amplification with the sensor, the influence of the ambient temperature on the circuit performance can be excluded. The MCU configures the ADC with a 32 Hz sampling frequency and 16 bits resolution to obtain the voltage signal. It converts the analog signal voltage value into a digital signal, and transmits it to the computer through Bluetooth.

To preliminarily verify the performance of the device in monitoring breathing, the airflow tube of the commercial ventilator (BY-Dreamy-B19) and the respiratory sensor were placed close together for testing, Figure 6d. The parameters of the ventilator were set to simulate the airflow of human breathing and a comparative study was conducted. The inspiratory pressure was set to 25 cmH_2_O, the expiratory pressure to 5 cmH_2_O, the temperature to 35 °C, and the test time to 35 s. Next, the inspiratory pressure was changed to 15 cmH_2_O, the expiratory pressure to 5 cmH_2_O, and the test duration and temperature remained unchanged. The test results are shown in Figure 6e, where the black line is the airflow generated by the ventilator, and the magenta and blue lines are the data collected by the sensors in the left and right channels of the device, respectively. The amplitudes of the signals of the two channels are significantly contrasted after changing the parameter. At the same time, the data collected by the two channels are basically consistent.

### 3.3. Monitoring of Respiration

A pilot study was performed on a non-professional healthy 23-year-old female volunteer (body mass 50 kg, and height 165 cm) to assess the feasibility of the wearable device for monitoring respiration. The participants in the respiratory monitoring experiment were investigators who volunteered to be subjects. The protocol for the study was approved by the Ethics Committee of Peking University Sixth Hospital. The volunteer sat quietly for ten minutes to ensure steady breathing. Then, we began to collect breathing signals for one minute and conducted five experiments.

**Figure 7:**
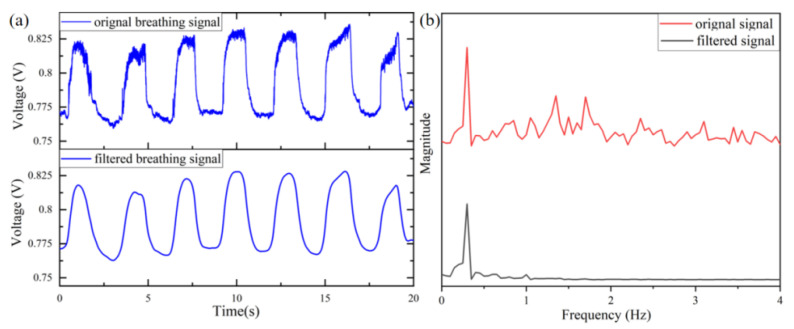
Breath signal recorded by the proposed system. (**a**) Original breathing signal and filtered breathing signal. (**b**) Magnitude analysis of the orignal signal and filtered signal.

To illustrate the respiratory waveform clearly, part of the original respiratory signal is shown in Figure 7a. Since the collected data are affected by noise and body movement artifacts, the signals need to be filtered. The results, after filtering in MATLAB, are shown in Figure 7a. Here, mainly Butterworth low-pass filtering was used to eliminate noise and baseline drift. The respiratory signal is filtered using a 10th order low-pass Butterworth filter with a cutoff frequency of 3 Hz. The magnitude of original signals and filtered signals are shown in Figure 7b, respectively. The respiratory rate is in the range of 0.2–0.5 Hz. It is clear that the device can monitor breathing signals effectively.

**Figure 8:**
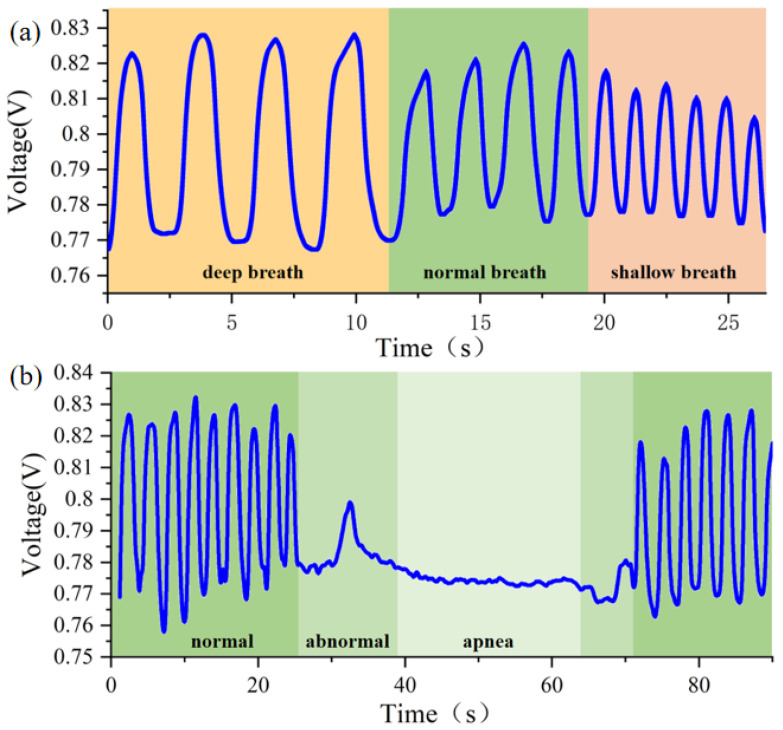
Breathing signal recorded by the proposed system. (**a**) Simulate different breathing patterns, including deep breathing, normal breathing, and shallow breathing. (**b**) The performance of the sensor in respiratory monitoring during normal breathing, abnormal breathing, and apnea.

The volunteer simulated breathing in different states, including normal breathing, deep breathing, shallow breathing, rapid breathing, and apnea. We obtained breathing waveforms of different frequencies and amplitudes. As shown in Figure 8a, the yellow part represents deep breathing, the green part is normal breathing, and the orange part is shallow breathing. The amplitudes of deep breathing are larger than shallow breathing. Shallow breathing is smaller in amplitude but with a greater breathing rate than deep breathing. In Figure 8b, normal breathing is indicated in green. During apnea, there is basically no change in the apparent amplitude. The light green part indicates abnormal breathing, and the frequency and amplitude of breathing are obviously different from normal breathing.

**Figure 9:**
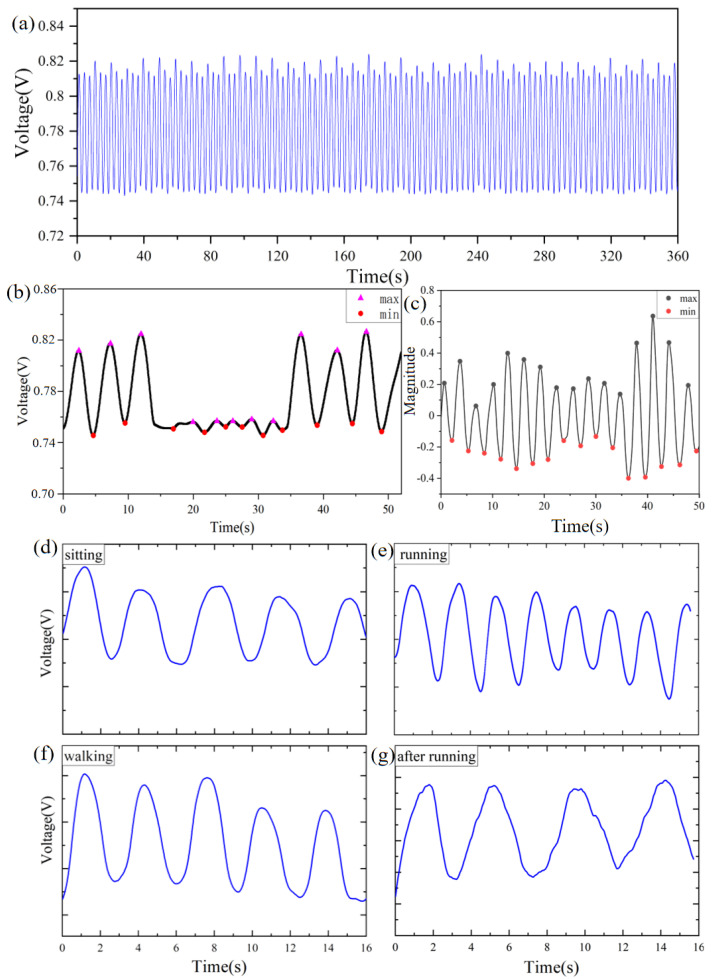
(**a**) Long−time measurement results (6 min). (**b**) Apnea respiration signal. (**c**) Hypopnea. The voltage changes of the sensor during different exercising. (**d**) Sitting. (**e**) Walking. (**f**) Running. (**g**) After running.

Monitoring sleep apnea is an important area, and sleep disorders require continuous breathing monitoring during sleep. The volunteer simulated sleep breathing, and we monitored long periods of normal sleep breathing signals for up to 6 min as shown in Figure 9a. The normal breathing signal during sleep is very stable. When apnea or hypopnea occurs, the signals change significantly, as shown in Figure 9b,c, respectively.

Here, we proposed a method for detecting apnea. Firstly, monitor the signal in the state of calm breathing, and calculate the amplitude of the respiratory waveform. Search the peaks and troughs of the respiration waveform, and the points where the respiration amplitude is significantly below the threshold. Conduct high-pass filtering of the signal with a cutoff frequency of 0.1 Hz and set the baseline to 0. Set the time window to 5 s and use increments of 1 s. Compare the extreme points in each time window to determine the peaks and troughs of waves. When the peak value is detected to be less than the threshold value or the valley value is greater than the threshold magnitude, the decreases in magnitude and duration are recorded. As shown in Figure 9c, a decrease in the breathing amplitude of more than 30% for more than 10 s is defined as sleep apnea hypopnea. When the distance between the peaks and troughs of the respiratory waves is reduced by 90% of the threshold of the normal amplitude and the duration is 10 s or more, it can be regarded as sleep apnea. Figure 9b shows the test result of respiratory signals under simulated apnea conditions for preliminary diagnosis. There is a period of apnea from 15 s to 34 s.

We measured people’s breathing in different exercise states as shown in Figure 9d–g. When sitting, the respiratory signal is relatively gentle, and the amplitude is small. The breathing rate of walking and the breathing rate of sitting are both about 0.3 Hz with little difference. When running, breathing speeds up and the frequency becomes greater, at about 0.5 Hz. Running is followed by the recovery process, for which the amplitude of the respiratory waveform is large, and the frequency reduces. The results demonstrate that wearing fPRS did not affect body movements and clearly distinguish between different types of body movements.

## 4. Conclusions

In summary, we propose a wearable respiratory sensor based on thermal sensitive materials with a perforated structure for respiratory monitoring. The respiratory sensor adopts a new structure and fabrication process, which improves the thermal conductivity of the sensor and solves the uncertainty in the process. The simulation results show that the perforated design will improve the dynamic range and the temperature sensitivity of the sensor. Because of the protection of the nasal air ducts, the sensors will not be directly interfered with by external factors such as environmental humidity and airflow; the sensors do not interfere with breathing.

In various respiration tests, the normal and abnormal breathing signals measured by the sensors show significant differences. Therefore, the proposed sensor is promising for sleep breathing monitoring and the diagnosis of respiratory disorders in the future.

## 5. Patents

This paper is based on some patents, such as the one named Nasal Respiratory Airflow Monitoring Device (ZL 2020 2 1943771.1).
